# Ureteral Closure Using Advanced Bipolar Vessel Sealing Devices During Laparoscopic Nephrectomy in Dogs and Cats: A Pilot Series of Clinical Cases

**DOI:** 10.3390/life14121681

**Published:** 2024-12-18

**Authors:** Przemysław Prządka, Bartłomiej Liszka, Kamil Suliga, Agnieszka Antończyk, Zdzisław Kiełbowicz, Dominika Kubiak-Nowak, Stanisław Dzimira, Adam Skalski, Ludwika Gąsior

**Affiliations:** 1 Department and Clinic of Surgery, Faculty of Veterinary Medicine, Wroclaw University of Environmental and Life Sciences, 50-366 Wroclaw, Poland; bartlomiej.liszka@upwr.edu.pl (B.L.); kamil.suliga@upwr.edu.pl (K.S.); agnieszka.antonczyk@upwr.edu.pl (A.A.); zdzislaw.kielbowicz@upwr.edu.pl (Z.K.); dominika.kubiak-nowak@upwr.edu.pl (D.K.-N.); ludwika.gasior@upwr.edu.pl (L.G.); 2 Department of Pathology, Division of Pathomorphology and Veterinary Forensics, Faculty of Veterinary Medicine, Wroclaw University of Environmental and Life Sciences, 50-366 Wroclaw, Poland; stanislaw.dzimira@upwr.edu.pl; 3 University Centre of General and Oncological Surgery, Wroclaw Medical University, 50-367 Wroclaw, Poland; adam.skalski@umw.edu.pl

**Keywords:** laparoscopy, surgery, animals, urology

## Abstract

Recently, laparoscopic nephrectomy has become more popular in veterinary medicine. In the majority of these procedures, vascular sealing devices (VSDs) are used. These allow for the closure of renal vessels with advanced bipolar coagulation. However, until now, closure of the ureter was performed with mechanical clips or suturing. There is a lack of information in the literature about the possibility of VSDs being used for ureter closure. This article presents the possibility of renal vessels and ureter closure in cats and dogs with vascular sealing devices. Laparoscopic nephrectomy in dogs and cats was performed entirely with VSDs. Patients with unilateral hydronephrosis qualified for the procedure. The nephrectomies were completely performed using a laparoscopic approach. Both renal vasculature and ureter were closed with VSDs. Additionally, two resected ureters from operated cats underwent histopathological evaluation. Among the operated animals, there were no postoperative complications or signs in the urinary tract. Histopathological evaluation of two cats’ ureters showed lumen closure on the coagulation places. Vascular sealing devices, during laparoscopic nephrectomy, allow for closure of not only the renal vessels but also ureters.

## 1. Introduction

A variety of unilateral renal disorders in dogs and cats can be treated by performing a nephrectomy. Traditionally, nephrectomy is performed via an open surgery approach. During this procedure, after a ventral median celiotomy, the kidney is dissected from its retroperitoneal attachments. The renal artery, renal vein, and distal ureter are ligated and dissected [1]. In veterinary medicine, nephrectomy can be used as a treatment for dogs and cats with renal disorders, including ureteral atresia, renal dysplasia, idiopathic renal hematuria, hydronephrosis, primary renal neoplasia, nephrolithiasis, polycystic kidney disease, chronic or unresponsive pyelonephritis, and trauma [1,2,3,4,5,6].

The published work that can be found on transperitoneal laparoscopic nephrectomy in research models or clinical patients within veterinary literature is limited [1,7,8,9,10]. Secure maintenance of homeostasis within surgery is crucial to safety in all operations.

For mechanical coaptation of 3 to 7 mm vessels within advanced laparoscopic nephrectomy, the most commonly used method are clips [11]. However, there is the possibility for clips to become dislodged during operative manipulation. If repeated applications are needed within the same area, they may also interfere with each other or with other stapling devices [11]. Moreover, due to their iatrogenic properties as a foreign body, clips can potentially erode into the surrounding tissue, which has been reported within the literature [12,13,14]. An alternative solution for vessel closure is ligation with suture, which can be more demanding technically and time consuming, especially during laparoscopic procedures [15]. Another option is the use of an advanced bipolar vessel sealing device. Using a combination of electrical currents and mechanical pressure, this device fuses vessel walls and creates seals. Coagulum is formed through mechanical pressure when collagen and elastin denature occur within the targeted tissue [15,16]. Within human surgery, evidence has shown that major branches of the renal vein, including the gonadal, adrenal, and lumber veins, can be sealed using vessel sealing devices [11]. At the same time, in animals, the renal vessels can be sealed using a vessel sealing device alone however the ureter is still closed using vascular clips [17]. To our knowledge, there is no information in veterinary literature about the use of a vessel sealing device in ureteral closure during nephrectomy in dogs and cats. The aim of this study was to evaluate the effectiveness and safety of ureteral closure using only a vessel sealing device during laparoscopic nephrectomy in dogs and cats.

## 2. Materials and Methods

### 2.1. Study Design and Patient Selection

This study was retrospectively approved by the ethics committee of Wroclaw University of Environmental and Life Sciences (Faculty of Veterinary Medicine Animal Welfare Advisory Team). All animal owners signed a consent form, after an explanation about the details of anesthesia and surgery procedures and their associated risks. Complete blood cell counts and biochemistry profiling were performed before the unilateral nephrectomy procedure.

The inclusion criteria for this study were dogs and cats with unilateral obstructive hydronephrosis requiring nephrectomy (Table 1). All operated animals were referred to the Department and Clinic of Surgery, Faculty of Veterinary Medicine, Wroclaw University of Environmental and Life Sciences in the years 2017–2022. All nephrectomies were performed by the first author in accordance with the applicable recommendations [1,18]. Animals qualified for the procedure had changes limited to one kidney, with the opposite kidney functioning properly based on ultrasound abdominal examination, morphological and biochemical blood tests and urinalysis results. Clinical observations were carried out for a minimum of one year after surgery.

### 2.2. Diagnostic Procedures

All animals underwent clinical and ultrasound examinations before the procedure to confirm the presence of moderate hydronephrosis with severe loss of renal parenchyma. Additionally, the ureters were examined using ultrasound from the kidney to the bladder. This allowed for measurement of the distended part cranially to the obstruction. Complete blood analysis (morphology and biochemistry), urinalysis, and urine culture were performed before surgery. Assessment of the functioning of the contralateral kidney using scintigraphy was not performed due to the lack of availability of such a test in the country (Poland).

### 2.3. Surgery

**Figure 1:**
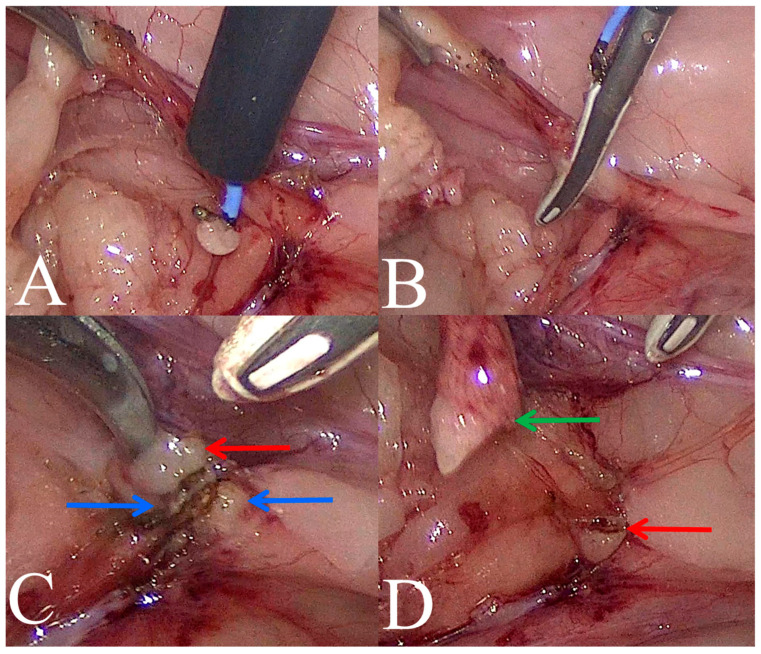
Intraoperative view of ureteral occlusion using a vessel sealing device (VSD) in a cat. (**A**)—dissection of the ureter; (**B**)—closing the ureter with a VSD; (**C**,**D**)—view of the ureter stump after its second closure and cutting using a VSD (green arrow—proximal part of the ureter, red arrow—ureteral incision line and the site of its closure with a VSD, blue arrow—distal part of the ureter).

All procedures were performed under general anesthesia. For the nephrectomy, the animal was placed in lateral recumbency with the affected kidney facing upward. The operated animal was covered with a sterile surgical drape immediately after antiseptic preparation in the surgical field. In all cases, an open technique with a 5 mm reusable trocar (through a longitudinal incision in the umbilicus) was used for establishing pneumoperitoneum using medical CO_2_. The optics were inserted after reaching an insufflation pressure in the abdominal cavity, which was 8 mmHg. Subsequently, under the control of the endoscope, two consecutive 5 mm and 10 mm diameter trocars were inserted in a triangular fashion. After gaining access to the abdominal cavity, the affected kidney was removed using a modified technique presented by Mayhew et al. [1,17]. The instruments used during the nephrectomy were Kelly dissection forceps (Karl Storz SE & Co. KG; Tuttlingen, Germany) and a vessel sealing device with an L-hook (ligasure™ retractable l-hook laparoscopic sealer/divider, Covidien, Minneapolis, MN, USA). To initiate dissection of the kidney, a vessel sealing device or monopolar electrosurgical L-hook were used to dissect the kidney from its retroperitoneal attachments. Next, the ureter was dissected out close to its insertion into the renal pelvis, which aided in the identification of the renal hilus by providing mild traction of the ureter. After renal vessels visualization and preparation, they were sealed and divided using the vessel sealing device alone. The remaining attachments of the kidney to the surrounding retroperitoneum were then sectioned using the vessel sealer. After the kidney was completely dissected, tension was placed on the proximal ureter. The ureter was cut distally beyond the level of the obstruction causing the hydronephrosis. Ureteral obstructions were identified during surgery based on macroscopic appearance—distention of the ureter cranially to the obstruction and its narrowing caudally to the obstruction. A vessel sealing device (VSD-ligasure™ retractable l-hook laparoscopic sealer/divider, Covidien, Minneapolis, MN, USA) was used to close the lumen of the ureter. In order to close the lumen of the ureter, the ureter was grasped in the VSD forceps, and the device was activated. After the completed coagulation cycle, the ureter was released without cutting the coagulated ureter tissues. Then, the same procedure was repeated 3–5 mm proximal to the previous coagulation site. After the completed coagulation cycle, the coagulated ureter tissues were cut with a knife present in the jaws of the VSD (Figure 1A–D). To facilitate resected kidney removal, its volume was reduced by puncturing with a needle introduced through the skin and collecting fluid within a syringe. Then, the resected specimen was placed into a specimen retrieval bag and removed through an enlargement of one of the instrument ports. The entire surgical site was re-examined for any ongoing hemorrhage and CO_2_ from the peritoneal cavity was removed. The trocar wound was closed using single intermittent sutures (monofilament 2/0; Dafilon; B. Braun, Rubi, Spain).

### 2.4. Follow-Up Evaluation

Ultrasound examinations of abdominal cavities and a complete physical examination were performed 24, 72 h, and two weeks after the procedure to detect any postoperative complications. Additionally, information was obtained by telephone from animal owners about the health status of the operated animals at least one year after the procedure.

The resected kidney and fragments of the ureter from the two last operated cases (cats) were fixed in 10% formalin, and after obtaining histopathological preparations, they were also stained with hematoxylin and eosin (HE). The histopathological slides were observed under an Olympus BX53 microscope coupled with an Olympus UC90 camera (Olympus, Tokyo, Japan). For acquisition, the cellSens Standard V1 software was used (Olympus, Tokyo, Japan).

### 2.5. Statistical Analysis

The median, range, numbers, and percentages of the overall results were reported for the animals affected by the variables of interest. The available software (Microsoft Corp., Version 16.56, Microsoft, Albuquerque, NM, USA) was used for all the calculations.

## 3. Results

### 3.1. Clinical Results

Laparoscopic nephrectomies were performed on the three dogs and four cats that were referred to our clinic with moderate hydronephrosis and severe loss of renal parenchyma due to ureter obstruction. All animals that qualified for the procedure participated in observations for at least a year after the surgery. The median age at the time of laparoscopic nephrectomy was 6 years (range, 4–8 years) for dogs and 6.5 years (range, 4–8 years) for cats (Table 1). The body weight of the operated dogs was from 4 kg to 32 kg, (mean weight 13.2 kg) and was from 3 kg to 4 kg (mean weight 3.5 kg) for the cats. Based on the abdominal ultrasound examination made for reasons unrelated to urological problems, hydronephrosis was detected incidentally in each case presented in this paper. Preoperative urine culture results did not reveal any urinary tract infection. Preoperative morphological and biochemical parameter blood tests and urinalysis tests were within the reference range.

All procedures were performed completely laparoscopically without the need for conversion. The renal blood vessels (artery and vein) were closed in all operated animals using VSDs without the presence of intraoperative and postoperative bleeding. The ureter in the operated animals were also closed using VSDs, and the closure of the ureter lumen was evidenced by the lack of urine leakage from the ureter stump on the side of the urinary bladder.

Physical examination performed 24, 72 h, and two weeks after the procedure did not reveal any postoperative abnormalities. None of the dogs that underwent surgery experienced major early or late complications. Follow-up ultrasound examinations performed 24, 72 h, and two weeks after the procedure did not show any postoperative complications, such as the presence of free fluid in the abdominal cavity or dilatation of the remaining ureter stump. Control blood tests performed 72 h after the procedure did not reveal any abnormalities in morphological and biochemical parameters, which were within the reference range.

According to the information obtained from the owners, at least one year after the surgery, none of the animals had urinary system symptoms, including those related to the nephrectomy performed. In one cat, a stone was found in the contralateral kidney during a control ultrasound examination.

### 3.2. Histopathological Results

**Figure 2:**
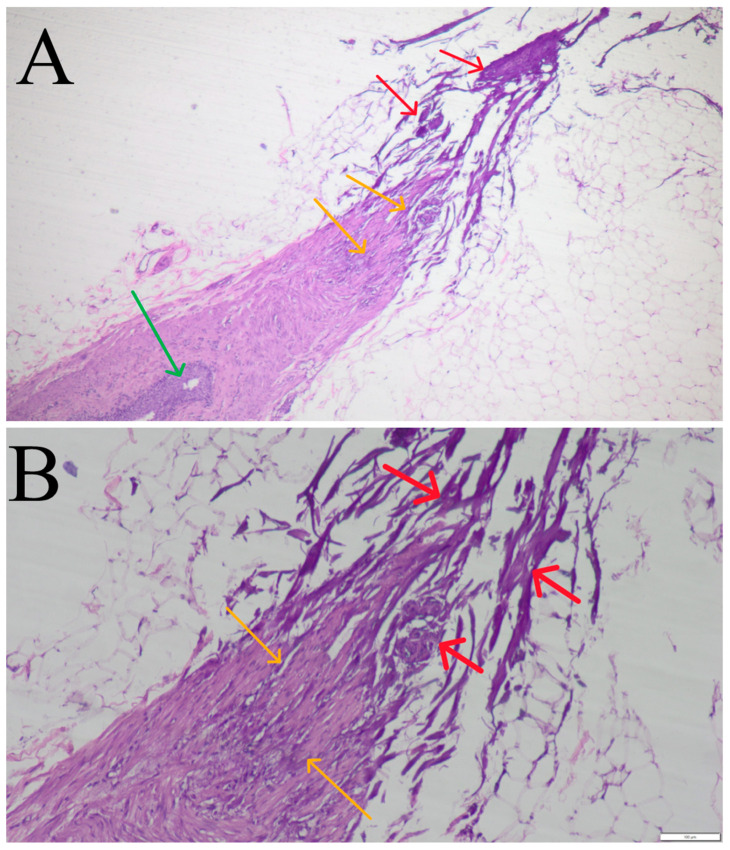
Histopathological images of the feline ureter occluded by vessel sealing device. (**A**)—Visible partial (among muscular layer—yellow arrows) and complete (at the dissected end of the ureter—red arrows) coagulation of the ureteral tissue and closure of its lumen. In the area of non-coagulated tissue, normal ureteral epithelium is visible (green arrow). Staining HE, magnification 40×. (**B**)—Visible partial (among muscular layer—yellow arrows) and complete (at the dissected end of the ureter—red arrows) coagulation of the ureteral tissue and closure of its lumen. Staining HE, magnification 100×.

Histopathological evaluation of the resected ureters revealed closure of the lumen by coagulation of the walls on its end (Figure 2A,B—red arrows). Blurring to the structure of the ureter’s muscular layer was observed. Initially, there were a few coagulated myocytes amongst the normal, unchanged muscular layer (Figure 2A,B—yellow arrows), as well as at the dissected end of the ureter where there were only coagulated myocytes visible (Figure 2A,B—red arrows). Here, only amorphous, highly basophilic band-like structures lying parallel to each other can be noted. Coagulated tissue did not bind together into one homogenous, amorphous compact mass but partially delaminated. A delicate fat tissue can be observed on the periphery. As is visible in Figure 2A, fragment of the epithelium before coagulation was not destructed (green arrow).

Histopathological evaluation was performed in two cats only. Owners of other patients did not consent for histopathological examination due to the economic reasons.

## 4. Discussion

In this paper, we present the possibility of safe and effective use of VSDs in ureteral closure during nephrectomy in selected animals with moderate hydronephrosis without enlargement of the distal ureter. In the presented study, there were no complications such as uroabdomen or hemorrhage. Uroabdomen following nephrectomy due to proximal ureteral leak is the most serious postoperative complication immediately following hemorrhage from the renal blood vessels. Laparoscopic nephrectomy was feasible in 7/7 cases (100%) without the need for conversion. It should be noted that laparoscopic closure of the ureter by VSDs was carried out in patients without distension of the distal part of the ureter. Mayhew et al. [1] stated that, in some patients, e.g., in extensive cancers, conversion to an open approach was necessary. Conversion to an open approach may be necessary in cases where the affected ureter is severely dilated or adhesions to the surrounding structures can be observed, particularly in situations where it is impossible to visualize the ureterovesicular junction [1]. In this pilot study, the ureter was closed directly distally from the obstruction without dissecting the ureter at the uretrovesicular junction. Short-term postoperative follow-up abdominal ultrasound did not reveal hydroureter in the remaining part of the resected ureter in any patient that qualified for the procedure using VSDs. According to the authors’ knowledge, the resecting of the ureter without its complete removal has not been described in veterinary literature before. The ureter was closed distally from the obstruction, where it was not dilated, and its diameter was corresponding to that in healthy animals. It should be noted that the biggest dog in this pilot study was 32 kg. Nephrectomy with complete ureter removal is traditionally recommended in small animals due to the fear of leaving a blind-ending ureteral stump, which may become a source of chronic infection and urine pooling [1]. Reviewing the literature, it can be noted that managing the distal ureter during laparoscopic nephro-ureterectomy is a recurring theme over the past decade [19,20]. Papers on what occurs to the ureteral stump post nephro-ureterectomy are limited [19,21]. Total removal of the ureter to the ureterovesicular junction in cases of urinary reflux is recommended in human pediatric medicine [22]. It should be noted that there is a possibility of complications related to the leaving of the ureteral stump post ne-phrectomy; however, there was no evidence within our patient group. The most common complication postoperatively during human surgery is infection of the remaining ureter including the ureteral wall, which shadows symptoms of pyelenophritis. There is the possibility that the infected ureteral stump may develop into an abscess. Suspicion of the ureteral stump abscess can be made if an oval cystic mass, adjacent to the bladder in ret-rovesical space, whose wall exhibits a contrast enhancement, which is found during computed tomography. Histopathological evaluation is required to confirm the diagnosis of empyema of the ureteral stump [22,23]. In post nephrectomy, a complication such as urinal reflux from the bladder requires the removal of the ureteral stump. A correlation between patients with high grade vesicoureteral reflux and complications which affected the stump were noted by Krarup and Wolf [24]. However, only 5% of cases exhibiting indications for stump removal were observed by Barroso et al. [25]. Moreover, ureteral stump removal was not necessary for any of the patients presenting with primary reflux into a single system. Therefore, there is a suggestion that the need for stumpectomy is linked to more complex cases. Advanced bipolar vessel sealing devices close vessels up to 7 mm in diameter with no risk of bleeding. In the vessels, the pressure is much higher than in the bladder; therefore, it is logical to use a VSD for closure of the ureter without the risk of urine leakage. Observations made within this pilot study confirmed this. Ureter closure was performed in two places adjacent to each other. First, the ureter was sealed with a VSD, then, after moving the device a few millimetres proximally, the ureter was sealed again and cut, allowing for kidney removal. Histopathological evaluation of resected ureters revealed their lumen sealed by coagulation. Observed changes in tissues resembled those caused by advanced bipolar coagulation within blood vessels [26]. The characteristic anatomical structure of the ureter opening into the bladder is an additional mechanism to prevent urine leakage. Ureters terminate at the cranial margin of the bladder neck after passing obliquely through its wall. The risk of urine reflux into ureters is reduced by this oblique intramural passage and intramural tension produced by pressure within the bladder [27]. The aforementioned mechanism probably does not work properly in cases of anatomical anomalies within the ureter and bladder connection and in patients after treatment of ectopic ureters. The authors of this pilot study decided to use a VSD for ureter closure when there were no signs of hydroureter in the sealing area. However, performing a complete ureter resection to the ureterovesical junction should be considered in cases of severe hydroureter [1]. Caution and further research are required in the use of VSDs for ureteral closure in patients with ureterovesicular junction abnormalities, or who had procedures for ectopic ureteral treatment performed in the past, as these cases can have an increased risk of urine reflux from the bladder to the ureters. This is an indication for removal of the whole ureter, up to the ureterovesicular junction. However, it requires longer laparoscopic dissection of the ureter, which sometimes can lead to additional complications, e.g., accidental damage of abdominal vessels or organs [28]. Another option is the intraoperative evaluation of stump leakage with retrograde pyelo-gram and/or cystogram. These procedures are used in human medicine for the evaluation of ureteral leakage [29]. In cases where postoperative free fluids within the abdomen are present, biochemical evaluation can be performed. A doubled increase in creatinine concentration in the fluid compared to the blood can suggest urine leakage [29].

Vessel sealing devices allow for the safe closure of blood vessels and thus reduce procedure times and the need to use other methods [1,11]. In the cases presented in this article, renal arteries and veins were closed using VSDs in all patients, the largest of whom was a 32 kg dog. Ligation of renal vessels can also be performed with laparoscopic hemoclips, extra- and intracorporeal ligation, or en bloc stapling [1,30]. There are advantages to using VSD for the closure of renal vessels. The use of the device allows for a reduction in procedure time and surgery as a whole [11]. It should be noted that the significance of time reduction in laparoscopic nephrectomy with VSDs in veterinary medicine needs to be evaluated. It also reduces the need of clips, staplers, or ligation with suture [11,12,13,31]. Within the patients of this pilot study, there were no intra- and postoperative complications. Follow-up abdominal ultrasound and blood results did not show any abnormalities in the early postoperative period. Additional information collected from the owner after at least one year post-surgery, also did not show any long term problems or complications. One cat developed a stone in the remaining kidney eight months after the nephrectomy. However, the diagnosis was made by a referring doctor during control examination and the cat was asymptomatic.

The occurrence of no postoperative complications is probably the result of the criteria for selecting patients for the procedure. Kidney removal with a VSD was performed in patients with hydronephrosis and hydroureter where dilation of the ureter was within its proximal part, no more than half the length. Furthermore, the patients who qualified for the procedure were diagnosed with hydronephrosis accidentally and showed no clinical signs related to the urinary system inclusive of blood and urine results.

The authors are aware of some limitations of this pilot study. The group of patients was relatively small and there were strict criteria for qualifying for the procedure—the ureters were closed in places where tissue appeared macroscopically normal in appearance without visible distention. Closing of the ureters with VSDs was not performed on the hydroureters or at the level of the ureterovesicular junction. In addition, analysis of the uroliths causing obstruction was not performed. The lack of urolith analysis was a result of the individual owners’ decisions despite the surgeon’s recommendations.

## 5. Conclusions

The results of the present study suggest that ureteral closure using an advanced bipolar vessel sealing device is a safe and effective alternative for closing the unextended ureter during nephrectomy. With this technique in selected cases, there is no need to use vascular clips, staplers, or ligatures for ureteral closing. The procedure allows the performance of nephrectomy using only Kelly dissection forceps, an advanced bipolar vessel sealing device, and a monopolar hook.

Partial removal of the ureter is possible. Leaving the non-dilated distal part of the ureter in a properly working ureterovesicular junction appears to be safe and does not lead to the development of postoperative hydroureter complications in a relatively short time after surgery. This subject needs a longer term of observation and critical assessment of each operated case.

## Figures and Tables

**Table 1:** Listing and additional information about operated animals.

Animal Number	Species	Breed	Sex	Age (Years)	Body Weight (kg)	Affected Kidney	Cause of Hydronephrosis
1	dog	Giant schnauzer	female	6	32	left	iatrogenic (OVH)
2	dog	Yorkshire Terrier	male	4	3.5	right	iatrogenic (ureterotomy)
3	dog	Maltese	male	8	4	left	ureteral stones
4	cat	European shorthair	male	6	3.5	left	ureteral stones
5	cat	European shorthair	female	7	4	right	ureteral stones
6	cat	European shorthair	female	5	3	left	ureteral stones
7	cat	Scottish fold cat	female	8	3.5	left	ureteral stones

## Data Availability

Data is contained within the article.
